# Supplementary material for: The Association Between Obstructive Sleep Apnea and Oral Function, Using the Korea National Health and Nutrition Examination Survey Data
Source: Healthcare (Basel). 2025 Jun 2;13(11):1323. doi: 10.3390/healthcare13111323 (PMC12154254; doi:10.3390/healthcare13111323)
Supplement: Supplementary file 1 [file healthcare-13-01323-s001.zip › healthcare-3609759-supplementary.pdf]

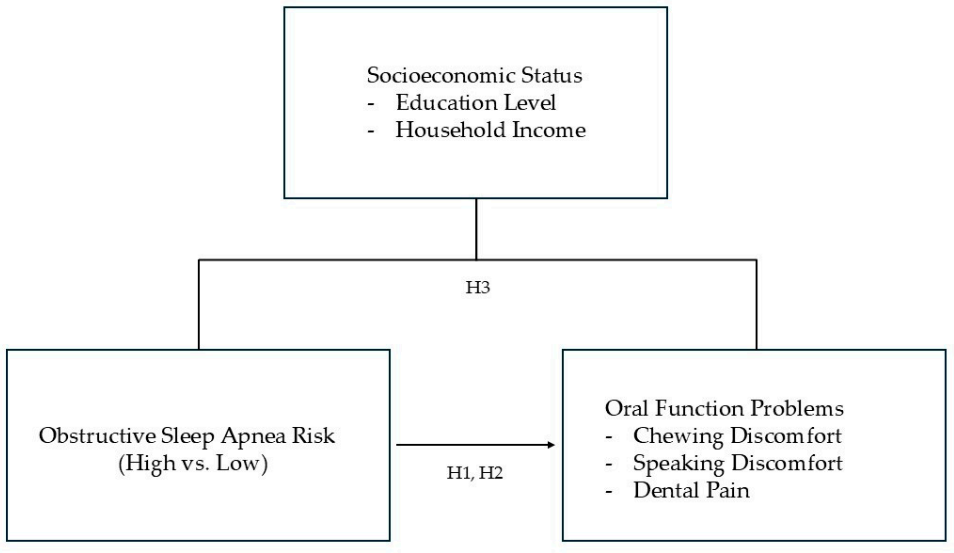

Supplementary Figure S1. Conceptual framework illustrating the hypothesized associations

H1: Obstructive sleep apnea (OSA) is significantly associated with oral function problems.

H2: Individuals with a higher risk of OSA are more likely to experience greater difficulties in chewing, speaking, and dental pain.

H3: These associations are more prominent among socioeconomically disadvantaged groups, particularly those with lower education or household income.

STOP	Yes	No
Is your **Snoring** louder than talking or loud enough to be heard through doors?		
Do you often feel **Tired** or sleepy during the day?		
Has anyone **OBSERVED** you stop breathin while you sleep?		
Do you have or are you being treated for high blood **PRESSURE**?		

BANG	Yes	No
**BMI** more than 30 kg/m^2?		
**Age** over 50 years old?		
**NECK** circumference >= 36.3 cm?		
Gender: **MALE**?		
Total Score		

High Risk : Yes 3-8

Low Risk: Yes 0-2

Note. BMI and neck circumference were adjusted for the Asian Population. BMI threshold was modified for 35 to 30, and neck circumference from 40 cm to 36.3 in, accordance with Korean and Asian standards.

Supplementary Figure S2. Components and Scoring Criteria of the STOP-Bang Questionnaire for OSA Risk Assessment

Supplementary Table S1. Effect Size Interpretation of Logistic Regression Results for Oral Function

Outcome Variable	OR	95% CI	Effect Size Interpretation
OSA (High vs. Low)			
Chewing Discomfort	1.365	1.121-1.662	Small
Speaking Discomfort	1.534	1.126-2.082	Small
Dental Pain	1.198	1.006-1.431	Small
Age (≥70s vs 40s)			
Chewing Discomfort	2.016	1.470-2.766	Medium
Speaking Discomfort	2.815	1.535-5.202	Medium
Dental Pain	0.673	0.494-0.901	Small Protective
Education Level (Very Low vs. High)			
Chewing Discomfort	2.078	1.595-2.707	Medium
Speaking Discomfort	3.308	1.884-4.905	Large
House Income (Q1 vs. Q4)			
Chewing Discomfort	1.942	1.510-2.496	Small
Speaking Discomfort	3.308	2.066-5.310	Large
Dental Pain	1.477	1.177-1.855	Small
Smoking (Current vs. Never)			
Chewing Discomfort	2.227	1.720-2.882	Medium
Speaking Discomfort	2.903	1.957-4.308	Medium
Dental Pain	1.507	1.194-1.901	Small
Sleeping Duration (≤6 hours vs. 7-9 hours)			
Chewing Discomfort	1.282	1.067-1.540	Small
HTN (Yes vs. No)			
Chewing Discomfort	0.741	0.619-0.888	Small Protective
Speaking Discomfort	0.606	0.455-0.807	Medium Protective

Note. Effect sizes were interpreted based on odds ratio (OR) magnitude using the following thresholds: OR < 2.0 was considered a *small* effect, 2.0 ≤ OR < 3.0 a *medium* effect, and OR ≥ 3.0 a *large* effect. Effect size interpretations were reported only for statistically significant associations ($p < 0.05$).

Among the significant findings, individuals at high risk for obstructive sleep apnea (OSA) showed greater odds of oral function problems compared to low-risk counterparts: chewing discomfort (OR = 1.365, 95% CI: 1.121–1.662), speaking discomfort (OR = 1.534, 95% CI: 1.126–2.082), and dental pain (OR = 1.198, 95% CI: 1.006–1.431), indicating small to medium effect sizes. Regarding education, those with very low education had higher odds of chewing (OR = 2.078) and speaking discomfort (OR = 3.308), reflecting medium to large effects. Lower education groups also showed elevated odds, including low education for chewing (OR = 1.559) and speaking (OR = 2.785), and moderate education for chewing (OR = 1.261) and speaking (OR = 2.033). As for income, participants in the lowest quartile (Q1) reported more chewing (OR = 1.942), speaking (OR = 3.308), and dental pain (OR = 1.477), suggesting small to large effects.

Supplementary Table S2. Effect Size Interpretation of Stratified Regression Results for Oral Function

Outcome Variable	Oral Health Outcome	95% CI	Effect Size Interpretation
Chewing Discomfort			
Inc: Q3 vs Q4	1.996	1.365-2.918	Small
Edu: Moderate vs High	1.983	1.357-2.897	Small
Talking discomfort			
Inc: Q3 vs Q4	5.207		Large
Inc: Q2 vs Q4	3.021	1.470-2.766	Medium
Inc: Q1 vs Q4	2.587	1.535-5.202	Medium
Edu: Moderate vs High	3.798	1.759-8.200	Large
Edu: Low vs High	3.750	1.657-8.488	Large
Edu: Very Low vs High	2.203	1.034-4.694	Medium
Dental Pain			
Inc: Q3 vs Q4	1.486	1.053-2.098	Small

Note. Effect sizes were interpreted based on odds ratio (OR) magnitude using the following thresholds: OR < 2.0 was considered a *small* effect, 2.0 ≤ OR < 3.0 a *medium* effect, and OR ≥ 3.0 a *large* effect. Effect size interpretations were reported only for statistically significant associations ($p < 0.05$).

Effect sizes were interpreted based on odds ratio magnitude to evaluate the strength of associations between socioeconomic indicators (education level and household income) and each oral function problem. For chewing discomfort, both moderate education level and third income quartile (Q3) showed statistically significant associations compared to their highest reference groups (OR = 1.983 and 1.996, respectively), and were interpreted as having small effect sizes. In the case of talking discomfort, the associations were generally stronger. Large effect sizes were observed in those with moderate and low education levels (OR = 3.798 and 3.750, respectively), as well as in those from the third income quartile (OR = 5.207). Moderate effect sizes were observed for the very low education group (OR = 2.203) and lower income quartiles (Q1 and Q2), suggesting a consistent gradient across socioeconomic indicators. Regarding dental pain, only one statistically significant association was found: individuals in the third income quartile (Q3) had a higher odds of reporting dental pain than those in the highest quartile (Q4), with a small effect size (OR = 1.486).
